# Comparative Analysis of Sociodemographic, Clinical Features, Laboratory Findings, and Treatment Protocols in Generalized and Localized Cutaneous Lichen Planus

**DOI:** 10.7759/cureus.79081

**Published:** 2025-02-16

**Authors:** Mustafa Urun, Gizem Karapinar, Yildiz Gursel Urun, Sezgi Sarikaya Solak

**Affiliations:** 1 Dermatology, Trakya University Faculty of Medicine, Edirne, TUR

**Keywords:** acitretin, classic lichen planus, cutaneous lichen planus, generalized lichen planus, hepatitis b & c

## Abstract

Background: Lichen planus (LP) is a chronic, inflammatory, and mucocutaneous disease that can present in various clinical forms, affecting the skin, mucosa, and appendages of the skin. A more extensive form of cutaneous LP, known as generalized cutaneous LP (GCLP), involves a significant portion of the body surface area. Because of the accompanying intense pruritus and difficulties with its treatment, the diagnosis and treatment of GCLP are gaining importance. In this study, we aimed to compare the characteristics of cutaneous LP with localized involvement and those of GCLP.

Methods: We retrospectively analyzed patients' sociodemographic characteristics (age, gender), clinical features (localization and duration of lesions), laboratory findings, treatments received, and relapse rates following treatment using electronic medical records obtained from our university’s digital registry system.

Results: Among the patients with cutaneous LP, 24.7% (n=46) had localized cutaneous LP (LCLP), and 22.5% (n=42) had GCLP. Involvement of the trunk and flexural regions was higher among the patients with GCLP than LCLP (p<0.001 and p=0.012, respectively). Hypertriglyceridemia and hepatitis B core antibody positivity were also observed more frequently in the patients with generalized than LCLP (p=0.005 and p=0.016, respectively). Narrowband ultraviolet B and acitretin were more effective in treating patients with GCLP (p<0.001 and p=0.001, respectively). When the relapse rates in both LP groups were compared, relapses were more frequent in the patients with GCLP (p=0.022).

Conclusions: The lesion localization, treatment needs, and relapse rates of patients with GCLP differ from those of patients with LCLP.

This article was previously presented as an e-poster at the 32nd National Turkish Dermatology Congress on November 24, 2024.

## Introduction

Lichen planus (LP) is an idiopathic subacute or chronic inflammatory disease of the skin, mucosa, and skin appendages [1]. Although the exact etiology of lichen planus is unknown, autoimmune, neuropsychiatric, hepatopathic, and metabolic mechanisms have been implicated [2]. The classic presentation of cutaneous lichen planus includes red to brown, violaceous, polygonal papules that are flat-topped, slightly scaly, and intensely pruritic [3]. The location of the lesions is usually symmetrical. The flexural surfaces of the forearms, wrists, and ankles; the dorsal surface of the hands; the shins; the trunk; and the sacral region are frequently involved [4].

Most cutaneous LPs resolve spontaneously within 6-18 months. The goal of treatment is to accelerate the healing of lesions and reduce itching [5]. Cutaneous LP is generally managed according to the location and severity of lesions and is largely dependent on clinical experience [6]. Topical steroids and tacrolimus are the first-line therapeutic options and are supported by a high level of evidence in the treatment of LP. Systemic steroid treatment is recommended for patients with severe and extensive lesions or those who do not respond to topical steroids [7]. In patients with GCLP in whom these treatment options fail, narrowband ultraviolet B (NB UVB) and acitretin stand out as good alternatives [8,9].

Patients with involvement of more than 20% of the body surface area (BSA) are considered to have GCLP [8]. Although GCLP typically manifests clinically as classic cutaneous LP lesions in the form of violaceous, flat-topped papules and plaques, it can also present as a generalized infiltrated exanthema of the skin with the coalescence of these lesions [3]. The treatment of GCLP is more challenging than that of other types of LP [10].

This study presents the sociodemographic and clinical characteristics, treatment protocols, and relapse rates of patients with LCLP and GCLP. Given the limited literature on GCLP, this study aims to (1) provide clinicians with information about GCLP, (2) identify comorbidities that may accompany this disease, (3) review the treatment options needed by patients with GCLP, and (4) compare the clinical features of patients with LCLP and GCLP.

## Materials and methods

This study was performed by retrospectively analyzing the records of patients who were admitted to the Department of Skin and Venereal Diseases, Faculty of Medicine, Trakya University, between January 2010 and January 2024, and who had been clinically and histopathologically diagnosed with LP. The records of 186 LP patients were analyzed in total. Subtypes of LP were classified based on criteria established in previous studies [11,12]. In our study, 51.6% of the patients had cutaneous LP (n=96), 32.2% had only oral LP (n=60), and 16.1% had only appendageal LP (n=30) (see Appendices). The study was approved by the Local Ethics Committee of Trakya University Faculty of Medicine (approval number: 07/07, date: 15.03.2024).

The clinical data were obtained from electronic medical records in the university’s digital registry system. The patients' sociodemographic characteristics (age, gender), clinical features (localization and duration of lesions), laboratory findings, treatments received, and relapse rates following treatment were analyzed. The patients whose lesions were localized to the wrists, forearms, distal lower extremities, and presacral regions were considered to have cutaneous LP with typical involvement sites [13], and patients with lesions involving more than 20% of the BSA were considered to have GCLP [8]. The flexural regions evaluated were the axilla, the inguinal fold, the gluteal cleft, the limb flexors, and the submammary region [6]. The disease duration was analyzed within three categories, namely, 0-3 months, 4-12 months, and over 12 months, in line with the study of Parihar et al. [1]. When evaluating the frequency of use of narrowband ultraviolet B (NB-UVB) and other conventional treatments (acitretin, methotrexate, hydroxychloroquine, isotretinoin), all patients were required to be unresponsive to treatments such as topical and/or systemic steroids and/or topical tacrolimus. Relapse was defined as the recurrence of active cutaneous lesions after initial healing, as stated in the study by Atzmony et al. [14]. Patients with insufficient histopathological findings, those diagnosed with drug-related LP, and patients whose records did not have all the data required for our study were excluded from the study.

The data were analyzed using IBM Corp. Released 2013. IBM SPSS Statistics for Windows, Version 22.0. Armonk, NY: IBM Corp. In descriptive analyses, frequency data were given as number (n) and percentage (%), and numerical data were given as arithmetic mean±standard deviation (SD), minimum-maximum (min-max). The distribution of categorical data was examined using Pearson Chi-square and Fisher Exact tests.

The distribution of numerical data was examined with the Shapiro-Wilk test. The distribution of numerical data in two independent groups that did not comply with normal distribution was evaluated with the Mann-Whitney U test. The statistical significance level for all tests was accepted as p<0.05.

## Results

**Figure 1:**
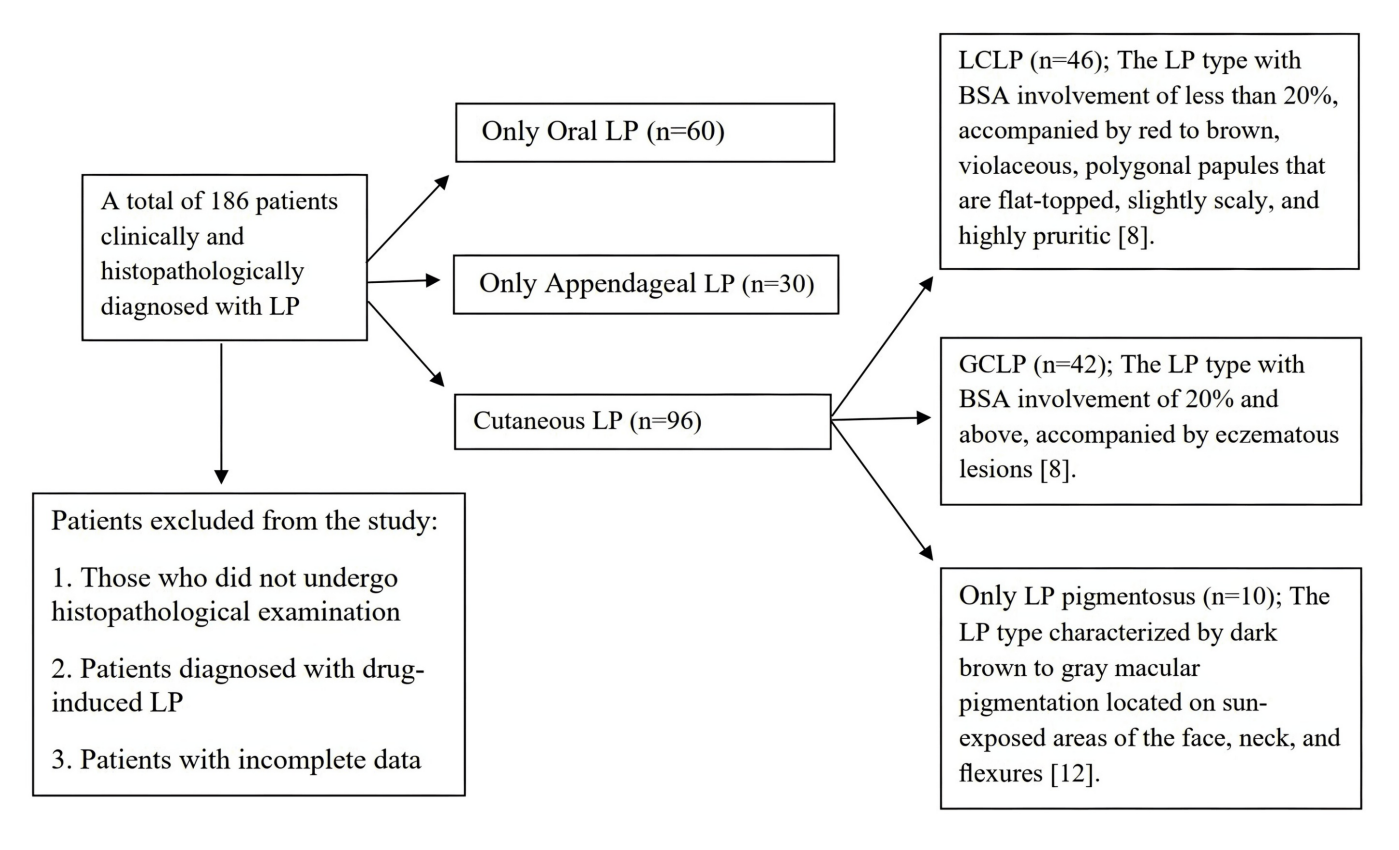
Flowchart illustrating the selection of the study sample LP: Lichen planus, LCLP: Localized cutaneous lichen planus, GCLP: Generalized cutaneous lichen planus, BSA: Body surface area

Among the patients diagnosed with cutaneous LP, 24.7% (n=46) had LCLP, 22.5% (n=42) had GCLP, and 10.4% (n=10) had only LP pigmentosus (Figure 1). The age and gender distributions were statistically similar in the patients with LCLP and GCLP (p=0.704 and p=0.580, respectively; Table 1).

**Table 1:** Comparison of sociodemographic characteristics of lichen planus patients with localized and generalized involvement The data has been represented as N and % values. Age is expressed as mean ± standard deviation. ^*^Mann-Whitney test for medians, ​​​​​​​^**^Pearson Chi-Square test for categorical variables, Statistical significance was considered at p <0.05. LCLP: Localized cutaneous lichen planus, GCLP: Generalized cutaneous lichen planus, SD: Standard deviation, n: Number of patients, %: Percentage of patients, Min: Minimum, Max: Maximum

		LCLP (n=46)	GCLP (n=42)	X^2^/Z	p-value
Age (Mean±SD; Min-Max)		49.65±16.39 (17-80)	48.05±17.80 (11-44)	-0.442	0.704^*^
Age, n (%)	0-19	2 (4.3)	3 (7.1)		-
	20-39	10 (21.7)	11 (26.2)		
	40-59	19 (41.3)	15 (35.7)		
	60-79	14 (30.4)	12 (28.6)		
	≥80	1 (2.2)	1 (2.4)		
Gender; n (%)	Female	35 (76.1)	34 (81.0)	0.371	0.543^**^
	Male	11 (23.9)	8 (19.0)		

When the patients in both groups were compared in terms of lesion localization, the rate of lesions in the trunk and flexural regions was significantly higher among the GCLP patients than the LCLP patients (p<0.001 and p=0.012, respectively). It was found that there was no statistical difference between the LCLP and GCLP groups regarding lesions located in typical areas of involvement (p=0.063, Table 2).

**Table 2:** Comparison of lesion localizations in lichen planus patients with localized and generalized involvement The data has been represented as N and % values. ^*^Pearson Chi-Square test and ^**^Fisher’s Exact test for categorical variables. Statistical significance was considered at p <0.05. ^§^The flexural regions evaluated were the axilla, the inguinal fold, the gluteal cleft, the limb flexors, and the submammary region. LCLP: Localized cutaneous lichen planus, GCLP: Generalized cutaneous lichen planus, n: Number of patients, %: Percentage of patients

Lesions Localizations (n, %)	LCLP (n=46)	GCLP (n=42)	X^2^	p-value
Upper extremity	36 (78.3)	37 (88.1)	1.305	0.305^*^
Lower extremity	36 (78.3)	39 (92.9)	3.020	0.082^*^
Trunk	10 (21.7)	36 (85.7)	35.146	< 0.000^*^
Flexural^§^	1 (2.2)	8 (19.0)	6.631	0.013^**^
Face/Neck	3 (6.5)	5 (11.9)	0.714	0.475^**^
Genitals	4 (8.7)	2 (4.8)	0.576	0.678^**^
Oral Mucosal	15 (32.6)	21 (50.0)	2.488	0.115^*^
Nail	0 (0)	2 (4.8)	2.193	0.230^**^
Are the lesions located in typical involvement sites?	17 (39.5)	8 (18.6)	3.462	0.063^*^

When examining other CLP types accompanying LCLP and GCLP, although no statistical difference was found between the two groups, the most common type of CLP accompanying GCLP was LP pigmentosus. (p=0.485, Table 3).

**Table 3:** Other types of cutaneous lichen planus accompany localized and generalized cutaneous lichen planus ^*^Fisher Exact Test. ^**^Other types of cutaneous LP accompanying LCLP or GCLP are as follows: 4 patients with palmoplantar LP, 3 patients with vesiculobullous LP, 3 patients with hypertrophic LP, and 1 patient with atrophic LP [11]. CLP: Cutaneous lichen planus, LCLP: Localized cutaneous lichen planus, GCLP: Generalized cutaneous lichen planus, n: Number of patients, %: Percentage of patients

		LCLP (n=46)	GCLP (n=42)	X^2^	p-value^*^
Other CLP types accompanying LCLP and GCLP n (%)	LP Pigmentosus	0	8 (47.1)	1.626	0.485
	Others^**^	2 (100.0)	9 (52.9)		

**Figure 2:**
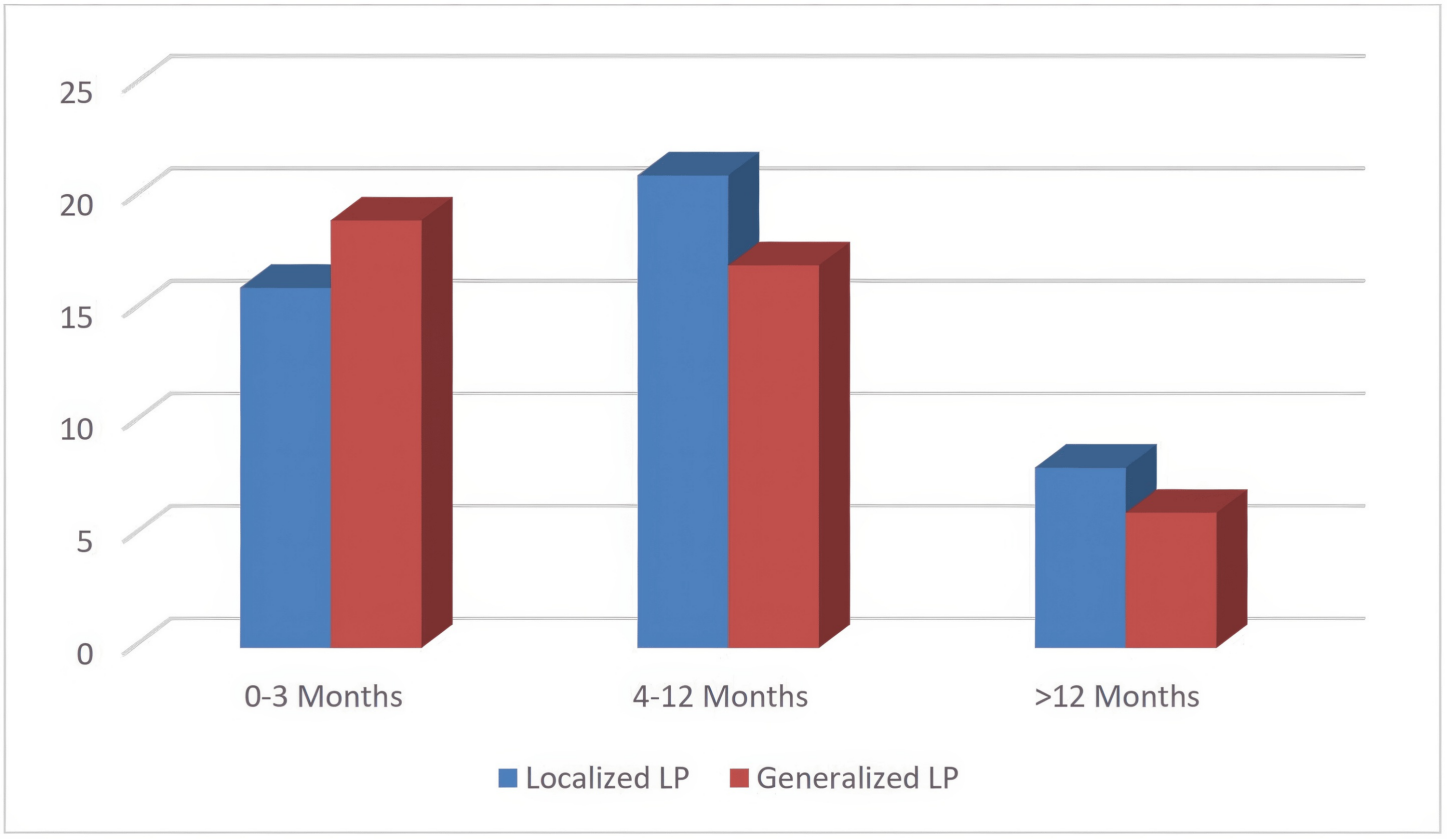
Distribution of lesion durations in localized and generalized lichen planus patients* The data has been represented as N values. ^*^Pearson Chi-Square test for categorical variables (p=0.577). Statistical significance was considered at p <0.05. ​​​​LP: Lichen planus

No statistically significant differences were found between the three categories of disease duration. The disease duration was 4-12 months in 45.7% (n=21) of LCLP patients and 0-3 months in 45.2% (n=19) of GCLP patients (p=0.577, Figure 2).

Evaluation of laboratory parameters revealed significantly higher rates of hypertriglyceridemia and hepatitis B core antibody (anti-HBc) positivity in GCLP patients than in LCLP patients (p=0.005 and p=0.016, respectively; Table 4).

**Table 4:** Comparison of laboratory findings in lichen planus patients with localized and generalized involvement The data has been represented as N and % values. ^*^Pearson Chi-Square test and ^**^Fisher’s Exact test for categorical variables. Statistical significance was considered at p <0.05. LCLP: Localized cutaneous lichen planus, GCLP: Generalized cutaneous lichen planus, ANA: Antinuclear antibody, TSH: Thyroid-stimulating hormone HBsAg: Hepatitis B surface antigen, Anti-HBc: Hepatitis B core antibody, Anti-HBs: Hepatitis B surface antibody, Anti-HCV: Hepatitis C antibody

Laboratory findings (n, %)	LCLP (n=46)	GCLP (n=42)	X^2^	p-value
Hypertriglyceridemia	8 (17.4)	19 (45.2)	8.005	0.005^*^
High HDL Cholesterol	10 (21.7)	16 (38.1)	2.612	0.106^*^
High Fasting Blood Sugar	18 (39.1)	12 (28.6)	1.256	0.262^*^
ANA Positivity	5 (10.9)	4 (9.5)	0.059	1.000^**^
High TSH Levels	4 (8.7)	3 (7.1)	0.090	1.000^**^
Anti-Thyroid Peroxidase Antibodies Positivity	4 (8.7)	6 (14.3)	0.622	0.509^**^
Anti-Thyroglobulin Antibody Positivity	3 (6.5)	2 (4.8)	0.146	1.000^**^
HBsAg Positivity	1 (2.2)	1 (2.4)	0.002	1.000^**^
Anti-HBc Positivity	4 (8.7)	12 (28.6)	5.607	0.016^*^
Anti-HBs Positivity	21 (45.7)	18 (42.9)	0.127	0.721^*^
Anti-HCV Positivity	2 (4.3)	0	1.911	0.495^**^

When the treatments for the two groups were compared, we observed that NB-UVB and acitretin treatments were more frequently chosen for patients with GCLP compared to those with LCLP (p<0.001 and p=0.001, respectively, Table 5).

**Table 5:** Comparison of treatment methods and relapse rates among patients with localized and generalized lichen planus The data has been represented as N and % values. Relaps time is expressed as mean ± standard deviation. ^*^Pearson Chi-Square test and ^**^Fisher’s Exact test for categorical variables. ​​​​​​​^***^Mann-Whitney test for medians. Statistical significance was considered at p <0.05. LCLP: Localized cutaneous lichen planus, GCLP: Generalized cutaneous lichen planus, n: Number of patients, %: Percentage of patients, NB UVB: Narrowband Ultraviolet B, SD: Standard deviation, Min: Minimum, Max: Maximum, LP: Lichen planus.

		LCLP (n=46)	GCLP (n=42)	X^2^/Z	p-value
Treatment, n (%)	Systemic Steroid (0.5-1 mg/kg/day for at least 3 weeks)	4 (8.7)	8 (19.0)	1.885	0.170^*^
	NB UVB (2 times a week)	1 (2.2)	14 (33.3)	14.736	<0.001^*^
	Methotrexate (15-25 mg/hafta)	0	1 (2.4)	1.084	0.483^*^
	Acitretin (25-35 mg/day)	5 (10.9)	17 (40.5)	9.915	0.002^*^
	Hydroxychloroquine (200 mg/day)	0	1 (2.4)	1.084	0.483^**^
	Isotretinoin (20 mg/gün)	1 (2.2)	1 (2.4)	0.002	1.000^**^
	Topical Tacrolimus	13 (28.3)	18 (42.9)	1.848	0.174^*^
	Topical Steroid	41 (89.1)	39 (92.9)	0.090	1.000^**^
Relaps, n (%)		1 (2.2)	8 (19.0)	6.808	0.022^**^
Relaps time (month), (mean±SD; Min-Max)		60.00	28.00±30.56 (3-62)	-0.603	0.667^***^

GCLP patients experienced relapses more frequently than LCLP patients when their relapse rates were compared (p=0.022).

## Discussion

Lichen planus can occur at any age but is most commonly observed in individuals in their third to sixth decades of life [11]. In our study, LP was most frequently observed in both LCLP and GCLP patients aged 40-59 and 60-79 years. This suggests that age is not a significant factor in the diagnosis of GCLP. The gender distribution among adults with cutaneous LP is not clear [11]. In a study conducted in 2022, it was reported that LP was more common in women [15]. Similarly, in another study in which GCLP patients were evaluated, a female predominance was apparent [8]. The data in our study support the findings in the literature. 

In case reports about the sites of involvement in patients with GCLP, extensive extremity and trunk involvement has been observed [16,17]. In our study, we found that trunk and flexural region involvement was more common in patients with GCLP than in those with LCLP. When comparing the presence of other lichen planus subtypes in LCLP and GCLP patients, it was seen that LP pigmentosus was more frequently associated with GCLP. Flexural regions are more frequently affected in LP pigmentosus disease [18]. This may explain the higher incidence of flexural involvement in GCLP patients. Clinicians should pay particular attention to trunk localization when diagnosing patients with GCLP.

Approximately one-third of cutaneous LP patients are reported to develop a widespread rash, which may appear after a week or longer and reach its maximum spread within 2 to 16 weeks [11]. Parihar et al. [1] reported that the disease duration varied in the patients with LP in their study. The disease duration in our study showed that lesions in most GCLP patients emerged within three months of symptom onset. Based on the literature, GCLP appears to have a more acute disease course, but further studies are needed on this subject.

Although the immunopathogenesis of LP is not yet fully defined, current hypotheses suggest that the condition is a result of T cell-mediated immunity or an autoimmune reaction against either endogenous or exogenous antigens [19]. The hypothesis suggests that the resulting chronic inflammation plays a role in disrupting lipid homeostasis [20]. Dyslipidemia has also been found to be more common in patients with LP [21]. The widespread cutaneous lesions in GCLP patients, coupled with resistance to first-line treatments, suggest a more severe inflammatory process in this subgroup. As a result, patients with GCLP experience chronic and severe inflammation, along with elevated triglyceride levels.

Several studies have pointed to an association between LP and cardiovascular disease or its risk factors. Higher fasting blood glucose and lipid levels have been reported in patients with LP [22]. Since our study did not include a healthy control group, it was not possible to interpret such data. In 2024, a study investigating the relationship between LP and metabolic syndrome (MS) emphasized that MS may be associated with oral mucosa involvement and LP severity [23]. Given the retrospective design of our study, a complete evaluation of all MS criteria was not feasible. However, triglyceride elevation, which is one of the diagnostic criteria of MS, was found more frequently in patients with GCLP than those with LCLP.

A relationship has been established between hepatitis C virus (HCV) infection and LP, and it is recommended that this test be performed in LP patients in regions with a high prevalence of HCV [15]. The presence of skin and mucosal manifestations in patients with LP who have HCV infection does not necessarily indicate a direct etiological association. HCV exhibits lymphotropism, which is linked not only to specific T-cell responses but also to the expansion of B lymphocytes. The stimulation of B lymphocytes results in auto-antibody production, which may contribute to various immunological alterations and B-cell proliferative disorders. As a result, autoimmune diseases such as diabetes mellitus and autoimmune thyroiditis are also frequently observed in LP patients [24]. It also suggests that the imbalance in oxidative stress in patients with LP, the disruption of the balance between oxidants and antioxidants, leads to a predisposition to HCV infections [25]. 

The study conducted by Doğan found a higher frequency of HBsAg positivity in patients with oral LP [26]. In a recent study, it has been reported that HBV infection is more frequent in oral LP patients [27]. In another LP study where subtyping was not performed, HBV antibodies were found to be more frequently detected in LP patients [28]. Patients who developed LP after hepatitis B vaccination have also been reported [29]. As a result, the relationship between LP and HBV has not been fully clarified.

There is insufficient data related to the frequency of HCV and HBV infections in patients with GCLP. In the study conducted by Turan et al. [13], 10 GCLP patients were evaluated before methotrexate treatment; no abnormality was detected in terms of hepatitis serology and hepatitis disease. In another case report, hepatitis serology was found to be negative [17]. A study conducted in 2024, including 31 patients with GCLP, reported no abnormalities in hepatitis serology during pre-treatment examinations [30]. When examining the data in our study, we found that anti-HBc positivity was more common among the patients with GCLP. We therefore surmise that patients with GCLP are more vulnerable to the hepatitis B virus. Although our study includes more patients than previous studies, larger studies are needed to clearly establish the relationship between GCLP and hepatitis.

LP is a chronic and relapsing disease [14]. A review of the literature reveals that patients with GCLP are often resistant to treatment and may require alternative therapies such as UVB phototherapy and methotrexate [8,13]. We observed in our study that GCLP patients commonly resist first-line treatments, yet respond favorably to NB UVB and acitretin.

In a study involving patients with generalized and recalcitrant LP, the relapse rate among those who initiated methotrexate treatment was reported as 40.6% [30]. In our study, this rate was found to be 19% in GCLP patients and was higher than in LCLP. We believe that the intense inflammation in patients with GCLP and their resistance to treatments may contribute to the increased risk of relapse. It should be kept in mind that relapses are more frequent in patients with GCLP, and different treatment options should be considered. 

Our study had some limitations: (1) a retrospective design, (2) a small study population, (3) the absence of a control group, and (4) a lack of information regarding the patients' sociodemographic (e.g., smoking) and clinical characteristics (e.g., body max index, pruritus severity) due to data loss.

## Conclusions

In our study, the trunk and flexor regions were more frequently involved, and the triglyceride levels were higher in patients with GCLP than those with LCLP. The former patients further needed NB UVB and acitretin treatments rather than classic LP therapy. LP also recurred earlier and more frequently in patients with GCLP than their counterparts with LCLP. This study provides a distinct advantage due to its larger sample size of GCLP patients. Furthermore, there is limited literature directly comparing the sociodemographic, clinical, and laboratory characteristics of LCLP and GCLP. Therefore, our data are of substantial significance in this context. To summarize, the clinical characteristics, treatment requirements, and relapse rates differ between GCLP and LCLP.
